# Temporal and Locational Adverbial Placement in Chinese–English and English–Chinese Translation: Corpus-Based Evidence from Advanced Chinese Learners of English

**DOI:** 10.3390/bs16101902

**Published:** 2026-10-14

**Authors:** Ran Meng, Ying Chen

**Affiliations:** 1 School of Computer Science and Engineering, Nanjing University of Science and Technology, Nanjing 210094, China; rmeng@njust.edu.cn; 2 School of Foreign Studies, Nanjing University of Science and Technology, Nanjing 210094, China

**Keywords:** adverbial placement, temporal adverbials, locational adverbials, learner translation, word order

## Abstract

This study examines how 35 second-year Master of Translation and Interpreting (MTI) students realized and placed temporal and locational adverbials in Chinese–English and English–Chinese translation. A learner translation corpus provided manually annotated data on adverbial type, form and position. Generalized linear mixed-effects models tested associations of realization and placement with form and position in the source text and associations of placement with form in the translation. In Chinese–English translation, temporal adverbials were more likely to retain their type when they were phrases or clauses rather than words and when they occurred initially rather than medially in the source text. In the translations, temporal words favored final position, whereas phrases and clauses favored placement before the predicate. For locational adverbials, neither realization nor placement differed significantly between canonical sentences and non-canonical sentences with locative–verb–subject (LVS) constructions. In English–Chinese translation, all temporal and locational adverbials realized as the same adverbial type occurred before the predicate. Locational adverbials occurring initially in the English source texts were usually placed initially in Chinese, whereas those occurring finally were more often placed medially. No comparable association was found for the placement of temporal adverbials. Learners’ realization and placement choices varied across adverbial types and translation directions.

## 1. Introduction

Compared with many other sentence constituents, adverbials allow greater positional flexibility in word languages. This flexibility is especially relevant to temporal and locational adverbials, whose placement can be used to study word order and to examine how learners organize time and place information across languages. Although Chinese and English are both predominantly Subject–Verb–Object (SVO) languages, they show different positional preferences for adverbials. Temporal and locational adverbials typically precede the predicate in Chinese but more often occur clause-finally in English, though initial position remains available in some contexts (Horová, 1976; Lü, 1999; Quirk et al., 1985; Ye & Jin, 2020). Chinese learners of English translating between the two languages may need to choose among possible positions for adverbials because source-text order, source-language preferences and target-language preferences may not coincide.

### 1.1. Temporal and Locational Adverbials in Chinese and English

The different positional preferences for temporal and locational adverbials can be understood in terms of linear organization. Chinese basic sentences follow the right-to-left (RL) expansion, whereas English basic sentences favor left-to-right (LR) expansion (Liu, 1992). With respect to weight, Chinese tends to place longer or more structurally complex constituents towards the beginning of a sentence, reflecting the front-weight principle (Jin, 1998), while English tends to place them towards the end, reflecting the end-weight principle (Quirk et al., 1972, 1985). These observations provide a broad basis for comparing the placement of temporal and locational adverbials in Chinese and English, but the relevant grammatical descriptions are not fully parallel across the two languages.

Research on temporal expressions in Chinese often examined time nouns, time adverbs, and other means of temporal reference (J. Y. Chen et al., 2025; Sun & Grisot, 2020), whereas research on English often discussed such expressions within the category of adverbials or adjuncts (Klumm, 2024). Studies of locational expressions in Chinese frequently focused on locative structures such as “*zài* + location” (Y. Xu, 2022), whereas studies on English often treated comparable expressions as adverbials, though some locative elements were analyzed as obligatory adverbials or complements (Huddleston & Pullum, 2002). In this study, temporal and locational expressions functioning as adverbials were classified as words, phrases, and clauses according to their forms. Their placement is examined in three positions, namely initial, medial, and final.

The positional preferences of temporal and locational adverbials are discussed separately below, with comparisons between Chinese and English for each type. In Chinese, temporal adverbials are typically placed before the predicate, either in sentence-initial position or in medial position, that is, between the subject and the predicate. Their positional preferences may vary across forms, including words, phrases, and clauses. At the word level, time nouns and time adverbs can occur in both initial and medial positions, but studies based on the CCL Corpus and the BLCU Chinese Corpus showed a general preference for medial, or post-subject, position (Yang, 2006; J. Y. Chen et al., 2025). Yang (2006) further found that the position of time adverbs is influenced primarily by the subject and also by syllabic length, with pre-subject position more closely associated with emphasis. J. Y. Chen et al. (2025) similarly showed that the preference for post-subject position is stronger for time adverbs than for time nouns, while pre-subject time nouns are more closely associated with topicalized contexts. At the phrasal level, Chinese temporal adverbials are commonly realized as noun phrases or prepositional phrases and are typically placed before the predicate. W. Li (2012) identified medial position as their default distribution, while D. Yu (2013) similarly noted that “preposition + temporal word” structures generally occurred before the verb when functioning as adverbials. At the clausal level, the preference for initial position becomes more evident. Although the notion of clause in Chinese does not map neatly onto the English concept of subordination, constructions such as “*zài … de shíhou (when/while)*”, “*dāng … de shíhou (when)*”, and clauses linked by “*yǐqián (before)*” or “*yǐhòu (after)*” are commonly treated as temporal clauses (Q. Zhang & Liu, 2022). Previous studies have consistently shown that such clauses tend to precede the main clause. Chao (1968) noted this tendency, and later corpus-based studies reported high proportions of temporal clauses occurring in initial position in both spoken and written Chinese (Wang, 1995, 2006; Yeh, 2000). Sentence-initial temporal clauses have also been associated with establishing a temporal frame for the following content (Wang, 2006). These studies show that temporal adverbials in Chinese are generally associated with preverbal positions, although the distribution varies across words, phrases, and clauses.

Temporal adverbials in English, however, allow greater positional flexibility. At the word level, their distribution often depends on the particular adverb and its function. Baker (1986) notes that temporal adverbs may occur in initial, medial, or final position, with medial position being relatively common. Adverbs expressing duration, however, tend to occur in final position. Ćetković (2014) found that “then”, “thereafter”, and “subsequently” occurred frequently after the subject in English police reports. By contrast, Fox (1993), as cited in Ćetković (2014), reported that post-subject “then” was relatively rare in both the written and spoken sections of the Bank of English. Choi (2025) further showed that the position of “now” varies with its function, tending toward final position when used as a temporal adverb and toward initial position when functioning as a discourse marker. Temporal adverbs in English do not show a consistent positional preference. At the phrasal level, temporal adverbials occur more often in initial and final positions than in medial positions. Baker (1986) noted that temporal adverbial phrases tend to appear at the beginning or end of the sentence. Klumm (2024) further showed that their distribution varies across genres, with clause-final placement more frequent in news reports and clause-initial placement more frequent in commentaries and student stories. At the clausal level, corpus-based studies have shown that English temporal clauses occur more often after the main clause than before it (C. H. Chen, 2004; Diessel, 2001, 2005; Ford, 1993; Y. Li & Wang, 2015). This tendency has been found across genres (Diessel, 2005; Klumm, 2024; Rezaee & Golparvar, 2016). Clause weight and sequencing iconicity also affect ordering, with clauses referring to earlier events more likely to precede the main clause than those referring to later events (Diessel, 2005, 2008). These studies suggest that temporal adverbials in English are more flexible in placement than their Chinese counterparts, with the tendency toward final position more consistent for clauses than for phrases.

Compared with temporal adverbials, locational adverbials have received less attention in research on adverbial word order, although previous studies still indicate clear positional differences between Chinese and English. In Chinese, locational adverbials are mainly associated with initial and medial positions. A common form is the prepositional phrase “*zài* + location”. J. Y. Fan (1982) showed that this structure may occur in three positions, but when functioning as an adverbial, it is typically preverbal. Initial position tends to establish a spatial frame for the event, whereas medial position more directly specifies the location of the action. Y. Xu (2022) further showed that different types of “*zài* + location” adverbials occupy different positions in multi-adverbial sequences, with those indicating the specific area of an action occurring closer to the main verb than those locating the whole event. A related pattern appears in the Mandarin Locative–Verb–Subject (LVS) construction (existence-appearance sentence), where the locative element precedes the verb and the postverbal noun phrase introduces the entity at that location (Y. Chen & Jing-Schmidt, 2014).

In English, locational adverbials are mainly associated with initial and final positions, with final position generally more common. In the 677 original English sentences examined by Horová (1976), SVO–Place–Time was the most frequent pattern, whereas patterns with clause-initial locational adverbials were comparatively rare. Medial position, particularly when interrupting the SVO sequence, was also uncommon. X. Zhang (2008) similarly noted that “in-phrases” functioning as temporal or locational adverbials in scientific English commonly occur after the verb. Studies of locative inversion further suggest that fronted locative elements are often associated with non-canonical word order and particular discourse functions rather than with the regular placement of locational adverbials (Bruening, 2022; Ojea, 2020). These studies suggest that Chinese locational adverbials are mainly associated with preverbal positions, whereas English locational adverbials more commonly occur in final position, with initial position often linked to particular structural or discourse conditions.

Overall, temporal and locational adverbials show broadly different positional distributions in Chinese and English, with preverbal placement more common in Chinese and final placement more common in English, although the details vary by adverbial type and form.

### 1.2. Temporal and Locational Adverbials in Learner English and Translation

From the perspective of second language acquisition, differences between the first and second languages may affect how learners use the second language. Structural contrasts may be more difficult to acquire than similar features, and learners may carry forms, meanings, or distributional patterns from their native language into the foreign language (Lado, 1957). Given the different positional preferences for temporal and locational adverbials in Chinese and English, Chinese learners of English may transfer Chinese word-order patterns into their English production. Previous studies of learner English further showed that, when several adverbial positions were grammatically possible, learners’ choices may also depend on L1 influence, adverb type, auxiliary use, and the surrounding syntactic context (Gledhill, 2005; Larsson et al., 2020, 2025; Osborne, 2008).

Research on Chinese learners’ adverbial placement in English has revealed positional preferences that differ from those of native speakers. B. Zhang and Koller (2015) reported a strong preference for sentence-initial position in Chinese ESL writing, although their study is not limited to temporal or locational adverbials. Differences from native-speaker preferences have also been found in adverbial clause ordering (Gries & Wulff, 2021). Studies specifically addressing temporal and locational adverbials have focused mainly on adverbial clauses of time. Chinese learners of English generally show a stronger preference for initial position than native speakers of English, and the strength of this preference may vary with the subordinator and clause structure (C. H. Chen, 2004; Fang, 2009; Y. Li & Wang, 2015). Research directly involving locational adverbials is much more limited. X. Li and Liu (2020) found that the placement of both temporal and spatial adverbials in learners’ production was affected by L1 conceptual transfer, with a stronger effect for temporal adverbials. Overall, previous research has provided considerable evidence on the placement of temporal adverbials, especially temporal clauses, whereas locational adverbial placement by Chinese learners of English has received relatively little attention.

The placement of temporal and locational adverbials becomes more complex in translation. Learners must coordinate the different positional preferences of the two languages, while their choices may also be influenced by the syntactic structure and word order of the source text. Although translations are expected to conform to target-language preferences, source-language interference has been detected in English translations from 16 European source languages across four language families (Dutta Chowdhury et al., 2021). J. Li and Hu (2024) found that English translations from Chinese showed evidence of source language shining through, retaining some characteristics of Chinese in their lexical, cohesive, and syntactic patterns. In Chinese–English translation by learners, source-text dependency distance has also been found to affect the syntactic complexity and quality of learners’ translations (Zhu & Wang, 2023). Source-language influence has also been found in English–Chinese translation. Zhu and Wang (2025) found significant correlations between English source sentences and Chinese translations in dependency distance and direction, providing evidence of the source language shining through at the syntactic level. Earlier corpus-based studies likewise showed that translated Chinese may retain lexical and grammatical features associated with English source texts (Xiao, 2010, 2015). More specifically, differences in word-order preferences between Chinese and English may affect how translators reorganize source-text structure. In Chinese–English sight translation, sentences whose word order differed from the target-language order were associated with more rereading, while in English–Chinese sight translation, word-order asymmetry was also found to increase cognitive load (Ma & Li, 2024; Ma et al., 2022). Although these studies concern sight translation, they provide further evidence that differences in word order between Chinese and English can affect translation in both directions. Such differences are particularly relevant to adverbials, because the two languages differ in their preferred placement of temporal and locational adverbials.

Previous studies have also discussed the repositioning of adverbials in Chinese–English and English–Chinese translation. In C-E translation, differences in adverbial order between the two languages may require positional adjustment rather than direct retention of source-text order (J.-F. Yu, 2000). In E-C translation, clause-final temporal, locational, and conditional adverbials in English are often moved to preverbal positions in Chinese (Z. Zhou, 1995). Similar repositioning has been reported for temporal adverbials in contract translation (G. Xu, 2002), as well as for English “in-phrases” functioning as temporal or locational adverbials (X. Zhang, 2008). Previous studies have mainly focused on adverbial repositioning in particular translation contexts or structures, leaving less fully explored how learners place temporal and locational adverbials across both translation directions and different forms of adverbial realization.

This study examines the realization and placement of temporal and locational adverbials in Chinese–English and English–Chinese translation by advanced Chinese learners of English. The learner translation corpus was compiled from source texts and translations produced by 35 second-year MTI students. To determine whether or not adverbials in the source texts were translated as adverbials of the same type, their realization in the translations was examined. The form of these adverbials was further coded as a word, phrase, or clause separately for the source texts and translations because an adverbial could take a different form during translation. Its position could also change during translation. Accordingly, position in the source text and placement in the translation were coded separately as initial, medial, or final. In addition, Chinese allows a non-canonical verb-subject (VS) order that is closely associated with locational expressions. In this structure, a locational adverbial typically occurs before the predicate, followed by the verb and a postverbal subject. This ordering is commonly referred to as a locative–verb–subject (LVS) construction. For locational adverbials, sentence type in the source text was therefore distinguished between canonical sentences and non-canonical sentences with LVS constructions. This distinction is therefore considered in examining the realization and placement of locational adverbials during translation. Therefore, the study addresses the following research questions:

(1) Do advanced Chinese learners of English translate temporal adverbials in the Chinese source text as temporal adverbials in the English target text? What placement do the learners choose in the sentence for temporal adverbials in Chinese–English translation?
(2) Do these learners translate locational adverbials in the Chinese source text as locational adverbials in the English target text? What placement do the learners choose in the sentence for locational adverbials in Chinese–English translation? Do realization and placement differ between canonical sentences and non-canonical sentences with LVS constructions?
(3) Do these learners translate temporal adverbials in the English source text as temporal adverbials in the Chinese target text? What placement do the learners choose in the sentence for temporal adverbials in English–Chinese translation?
(4) Do these learners translate locational adverbials in the English source text as locational adverbials in the Chinese target text? What placement do the learners choose in the sentence for locational adverbials in English–Chinese translation?

## 2. Materials and Methods

### 2.1. Corpus Construction

The data were drawn from the translation assignments and examinations of 35 second-year MTI students at Nanjing University of Science and Technology. The students were advanced Chinese learners of English with formal training in translation and had passed the College English Test Band 6 (CET-6). They were not permitted to use machine translation. No fixed time limit was imposed, and no teacher feedback was provided before submission. A learner translation corpus was compiled from the students’ translations and their corresponding source texts.

The corpus includes both Chinese–English and English–Chinese translation data, based on separate sets of Chinese and English source texts. The three Chinese source texts comprised a regulation for a sports competition (1066 characters; 23 sentences), an introduction to a historical and cultural site (1771 characters; 39 sentences), and a discussion of Chinese history (601 characters; 12 sentences). The three English source texts consisted of a guide to business culture (543 words; 36 sentences), an academic book preface (891 words; 30 sentences), and a cultural article on places in literary works (371 words; 15 sentences). Each of the six source texts was translated by all 35 students, yielding a total of 210 translations.

Temporal adverbials were expressions that served as modifiers of the clause or predicate by specifying temporal location, duration, frequency, or a temporal relation to another event or reference point. Temporal expressions occurring as focused constituents in cleft constructions were excluded. Locational adverbials were expressions that specified the location or spatial setting associated with the event, state, or situation described in the clause. They provided additional locational information rather than functioning as locative complements required to complete the predicate. Predicative locatives were excluded because the locational expressions in such constructions form part of the predicate, as in Chinese X *wèiyú* Y (“X is located in Y”). Under this functional definition, locative constituents preceding the verb in LVS constructions were also coded as locational adverbials because they served the same locational function. Canonical sentences and sentences with LVS constructions were analyzed separately.

In the Chinese source texts, 56 temporal adverbials, six locational adverbials in canonical sentences, and 12 locational adverbials in LVS constructions were identified, while the English source texts contained 24 temporal adverbials and 15 locational adverbials.

### 2.2. Annotation and Coding

Temporal and locational adverbials in the source texts and translations were manually annotated with a semi-automatic annotation tool developed in Microsoft Word 2016, using XML-style tags (G. P. Fan, 2001). The tag set drew on existing annotation practices in Chinese corpora, including the Peking University corpus and the Tsinghua Chinese Treebank (S.-W. Yu et al., 2002a, 2002b; Q. Zhou, 2004). The annotation had three nested layers: adverbial type, position, and form. The outer layer marked adverbial type, the middle layer marked position, and the inner layer marked form.

In the outer layer, temporal and locational adverbials were tagged as *tadv* and *sadv*, respectively, with *s* retained from the spatial labeling convention used in the reference annotation schemes. In the middle layer, position was coded as initial, medial, or final, following Quirk et al.’s (1985, pp. 490–500) classification of adverbial positions, which was adapted to the present corpus. These position labels were used directly as tags. Initial referred to the position before the subject. Medial referred to a position right after the subject and, where an operator was present, immediately after the operator. Final referred to a position following all obligatory elements in the relevant clause. An adverbial occurring at the end of its clause was therefore coded as final even when another clause followed within the same sentence. Where more than one adverbial occurred in the same sentence, each was annotated separately. In sentences or clauses without an overt subject, where the subject could not serve as a reference point, adverbial position was determined from the sentence structure and context. In the inner layer, form was coded as word, phrase, or clause, using the tags *t*, *tp*, and *tc* for temporal adverbials, and *s*, *sp*, and *sc* for locational adverbials. Form was determined independently for Chinese and English according to the syntactic structure of each expression. Expressions with internal modifiers were coded as a single phrase when they formed one syntactic constituent. Adverbials that formed separate syntactic constituents were annotated separately, even when they corresponded to a single expression in the other language. The complete tag set is shown in Table 1.

An example of the three-layer annotation is shown below:

(1)

Make the effort to learn a few words of Mandarin <tadv><final><tp>before your trip</tp></final></tadv>.

The annotated temporal and locational expressions in the source texts are presented by form and position in Table 2.

For each temporal or locational adverbial in the source texts, the corresponding learner translation was classified as a same-type adverbial, another type of adverbial, a non-adverbial expression, or an omission. The annotation was carried out by four native speakers of Chinese with formal training in translation. Each text was independently annotated by two of them. Any disagreements were discussed and resolved by consensus before the data were retrieved and analyzed. The original independent annotations were not retained separately, so inter-annotator agreement could not be calculated retrospectively.

### 2.3. Data Analysis

After annotation, the data for Chinese–English and English–Chinese translation were retrieved and analyzed separately. For the analysis of realization, the four categories of same-type adverbial, another type of adverbial, non-adverbial expression, and omission were collapsed into two groups. Same-type adverbials formed one group, and the remaining three categories formed the other. Placement analyses were limited to cases classified as the same type of adverbial. Statistical analyses were conducted in R 4.0.5 (R Core Team, 2021).

In Chinese–English translation, temporal and locational adverbials were analyzed separately. Binomial generalized linear mixed-effects models (GLMMs) were used, with crossed random intercepts for learners and individual adverbials in the source texts. The GLMMs for temporal adverbials are summarized in Table 3. Given the rarity of medial placement in the translation, initial and medial positions were combined as placement before the predicate, while final position was treated as placement after the predicate. Likelihood-ratio tests were used to assess the overall contribution of predictors in each of the three temporal GLMMs. Tukey-adjusted pairwise comparisons were conducted following a significant overall test of form, to determine which pairs of forms differed in placement. For Placement 2, given the small number of temporal adverbials in final position in the source texts, a sensitivity analysis was also conducted using only temporal adverbials in initial or medial position in the source texts and their corresponding translations.

For locational adverbials, the analysis additionally distinguished canonical sentences from sentences with LVS constructions. The six GLMMs are summarized in Table 4. Only the variable levels included in each analysis are shown in the tables. Levels not shown either did not occur in the corresponding data or the corresponding variables were not included in the analysis. Realization and placement were first examined in relation to sentence type in the source text and were then analyzed separately within the two sentence types. Given that all locational adverbials in LVS constructions occurred in initial position in the source text, sensitivity analyses were conducted for Realization 1 and Placement 1 by restricting the data to locational adverbials in initial position in the source text. Within LVS constructions, form in the source text was examined as the predictor, whereas within canonical sentences, position in the source text was examined because all locational adverbials were phrases. The realization model for canonical sentences initially produced a singular fit because the variance for learners was estimated at essentially zero. It was therefore re-estimated with a random intercept for individual adverbials in the source texts only, while retaining the same fixed-effect structure. For placement analyses, form in the translation was not included because locational adverbials in the translations were predominantly phrases, with too few words and clauses for reliable model estimation. Placement was coded in the same way as for temporal adverbials. Likelihood-ratio tests were used to assess the overall contribution of predictors in each of the six locational GLMMs.

In English–Chinese translation, temporal and locational adverbials were also analyzed separately. The three GLMMs for temporal adverbials are summarized in Table 5. The same analytical framework was applied as in the Chinese–English temporal analysis. No same-type temporal adverbials occurred in final position in the Chinese translations. Placement was therefore modeled as initial versus medial position. Given the small number of temporal adverbials in medial position in the English source texts, sensitivity analyses were conducted for the realization model and the second placement model (Placement 2) using only temporal adverbials in initial or final position in the source texts and their corresponding translations. Likelihood-ratio tests were used to assess the overall contribution of predictors in each of the three temporal GLMMs.

The two GLMMs for locational adverbials are summarized in Table 6. Form in the English source texts was not included as a predictor because all locational adverbials were phrases. No locational adverbials occurred in final position in the Chinese translations. Placement was therefore modeled as initial versus medial position. Likelihood-ratio tests were used to assess the overall contribution of predictors in the two locational GLMMs.

One of the three English texts, the academic book preface, contained a series of recurring initial “In Chapter X” expressions with similar structures. To assess whether or not this concentration affected the results, robustness analyses were conducted by adding English text as a categorical control variable and by repeating the analyses without the data from the academic book preface. Parametric bootstrap likelihood-ratio tests were also conducted.

## 3. Results

### 3.1. Temporal Adverbials in Chinese–English Translation

The 35 learners translated the 56 temporal adverbials in the source texts as temporal adverbials in 1617 instances, as other types of adverbials in 56 instances, as non-adverbial expressions in 155 instances, and omitted them in 132 instances. Realization as a temporal adverbial in the translation was associated with both form and position in the source text, with detailed results presented in Table 7. Likelihood-ratio tests showed significant overall associations for form, *χ*^2^ (2) = 17.91, *p* < 0.001, and position, *χ*^2^ (2) = 6.59, *p* < 0.05.

**Figure 1:**
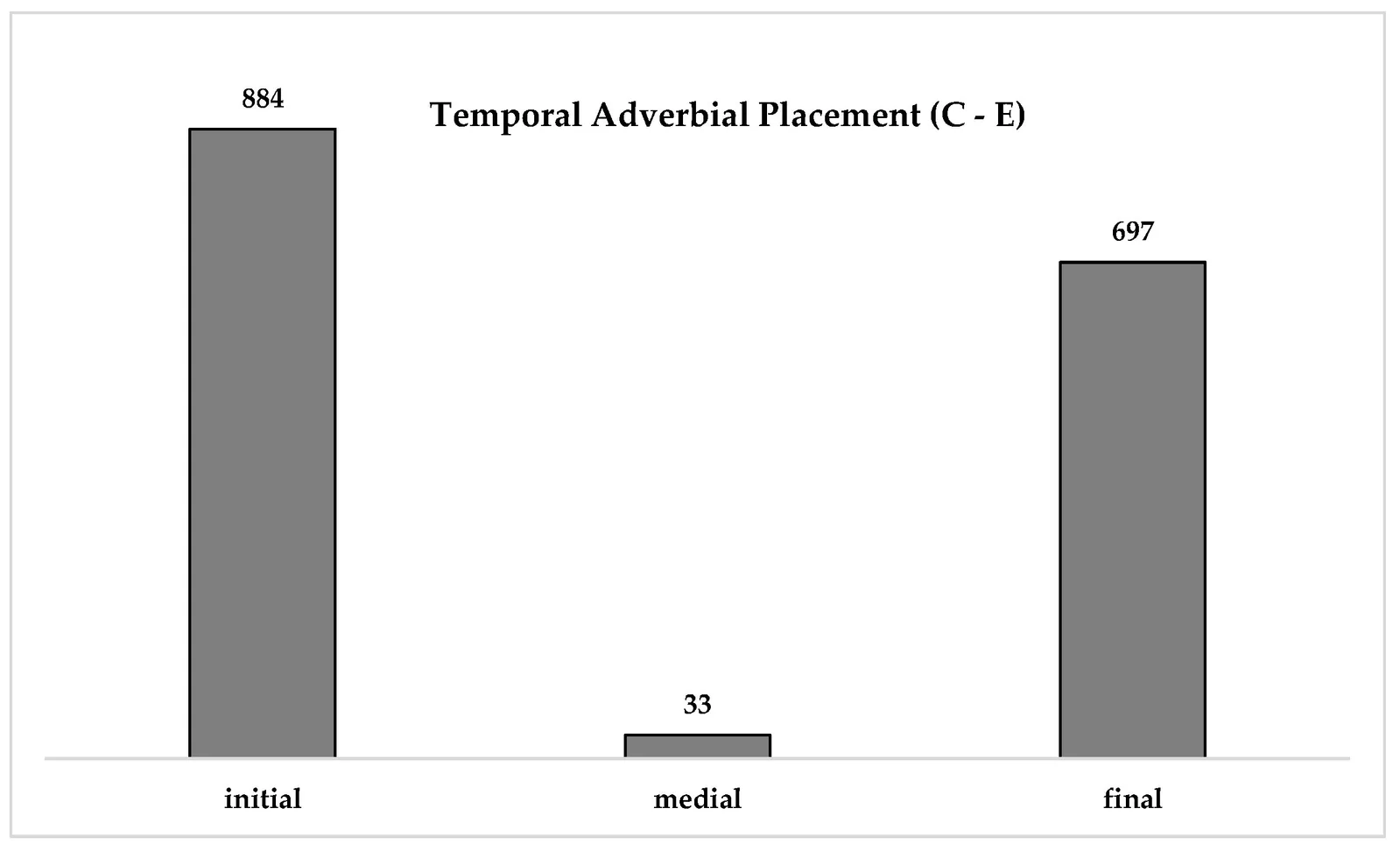
Distribution of temporal adverbials by position in Chinese–English translation.

Three of the 1617 instances were excluded from the subsequent analyses because their target form could not be reliably classified, leaving 1614 temporal adverbials for the placement analysis. The learners used 1614 temporal adverbials in their English translations, including 884 in initial position, 33 in medial position, and 697 in final position (see Figure 1). Temporal adverbials in initial and medial positions together accounted for 56.82% of the total, compared with 43.18% in final position.

**Figure 2:**
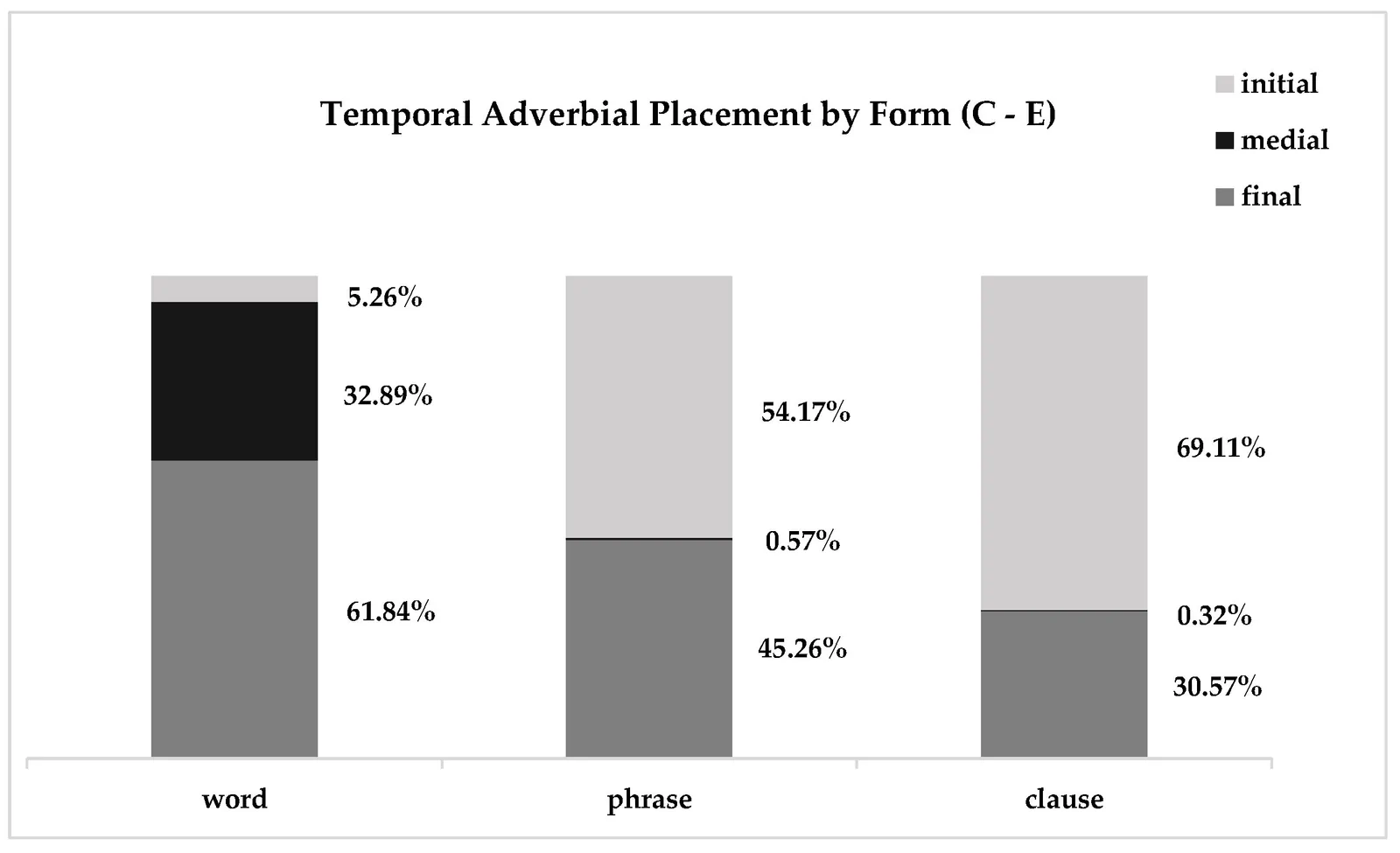
Distribution of temporal adverbials by form and position in Chinese–English translation.

As shown in Figure 2, the learners’ placement choices for temporal adverbials varied by form. Temporal words were most frequently found in final position (61.84%), followed by medial position (32.89%), while only 5.26% occurred in initial position. Temporal phrases occurred mainly in initial (54.17%) and final (45.26%) positions, with medial placement being rare. Temporal clauses showed the clearest concentration in initial position, with 69.11% appearing there.

Placement of temporal adverbials in the translation was associated with form in the translation, with detailed results presented in Table 8. A likelihood-ratio test showed that this overall association was significant, *χ*^2^ (2) = 9.33, *p* < 0.01. Tukey-adjusted pairwise comparisons showed that words had higher adjusted odds of pre-predicate placement than phrases (*OR* = 7.17, 95% CI [1.52, 33.93], *p* < 0.01), whereas the other pairwise comparisons were not significant.

Placement in the translation was not significantly associated with form in the source text, *χ*^2^(2) = 0.70, *p* = 0.704, but was significantly associated with position in the source text, *χ*^2^(2) = 21.75, *p* < 0.001. However, the comparison involving final position produced complete separation, and the main model did not converge normally. The sensitivity analysis yielded the same conclusion, with detailed results presented in Table 9. Temporal adverbials occurring in medial position in the source text had lower odds of pre-predicate placement than those occurring in initial position (*β* = −3.142, *SE* = 0.851, *OR* = 0.04, 95% CI [0.008, 0.23], *p* < 0.001).

### 3.2. Locational Adverbials in Chinese–English Translation

For the 18 locational adverbials in the source texts, the 35 learners translated them as locational adverbials in 275 instances, as other types of adverbials in 15 instances, as non-adverbial expressions in 282 instances, and omitted them in 58 instances. Of the 210 locational adverbials from canonical sentences, 125 (59.52%) were translated as locational adverbials, compared with 150 of the 420 instances (35.71%) from sentences with LVS constructions. Realization as a locational adverbial in the translation was not significantly associated with sentence type in the source text, with detailed results presented in Table 10. A likelihood-ratio test showed that the overall association was not statistically significant, *χ*^2^(1) = 2.83, *p* = 0.093.

The sensitivity analysis restricted to locational adverbials occurring in initial position in the source text likewise showed that realization as a locational adverbial in the translation was not significantly associated with sentence type in the source text, *χ*^2^(1) = 2.06, *p* = 0.151. Within sentences with LVS constructions, 58 of the 245 locational adverbials that were words in the source text (23.67%) were realized as locational adverbials in the translation, compared with 92 of the 175 phrases (52.57%). Realization as a locational adverbial in the translation showed no significant association with form in the source text within sentences with LVS constructions, *χ*^2^(1) = 3.36, *p* = 0.067. Within canonical sentences, 64 of the 105 locational adverbials occurring in initial position in the source text (60.95%) and 61 of the 105 occurring in medial position (58.10%) were realized as locational adverbials in the translation. Realization as a locational adverbial in the translation did not vary significantly by position in the source text, *χ*^2^(1) < 0.01, *p* = 0.977.

**Figure 3:**
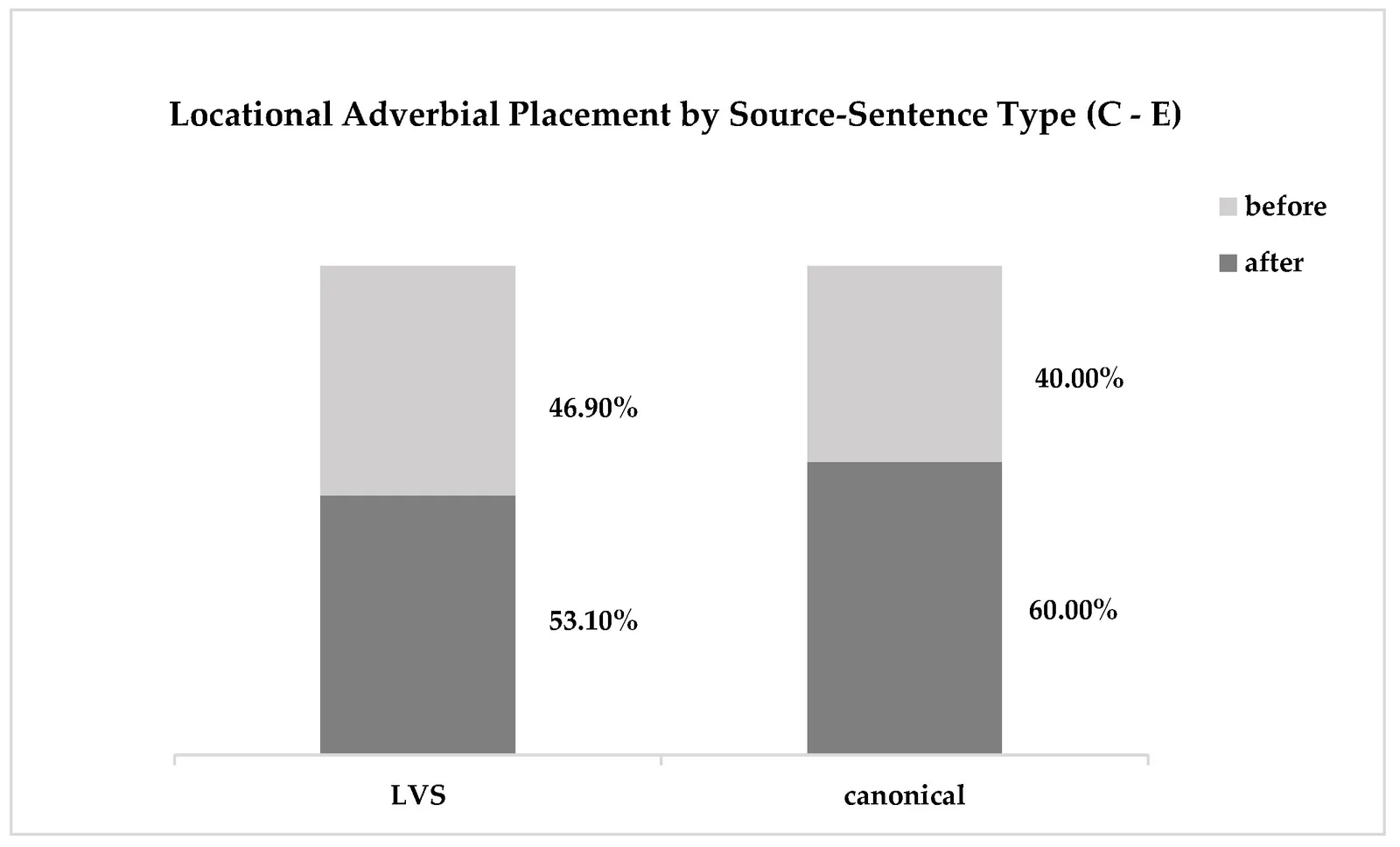
Distribution of locational adverbials by source-sentence type and position in Chinese–English translation.

Of the 275 same-type locational adverbial instances, 270 were included in the placement analysis. The other five were excluded because they involved either multiple corresponding adverbials in the translation or syntactically incomplete translations in which the adverbial position could not be determined. Among the 125 instances from canonical sentences, 50 occurred in initial position and 75 in final position, with none in medial position. Among the 145 instances from sentences with LVS constructions, 64 occurred in initial position, four in medial position, and 77 in final position. Given the rarity of medial placement, initial and medial positions were combined for the subsequent analysis as placement before the predicate, while final position was treated as placement after the predicate (see Figure 3). Placement before the predicate accounted for 46.90% of the LVS group and 40.00% of the canonical group.

Placement in the translation was not significantly associated with sentence type in the source text, *χ*^2^(1) = 0.16, *p* = 0.689. The sensitivity analysis restricted to locational adverbials occurring in initial position in the source text likewise showed no significant association between sentence type and placement in the translation, *χ*^2^(1) < 0.01, *p* = 0.990. Within canonical sentences, placement in the translation did not vary significantly by position in the source text, *χ*^2^(1) = 0.08, *p* = 0.782. Within sentences with LVS constructions, placement in the translation showed no significant association with form in the source text, *χ*^2^(1) = 0.15, *p* = 0.696.

Of the 270 locational adverbials included in the placement analysis, 255 (94.44%) were phrases, 12 were words, and three were clauses. Among the phrases, 145 (56.86%) occurred after the predicate, compared with six of the 12 words (50.00%) and one of the three clauses (33.33%). In the canonical group, 123 of the 125 locational adverbials were phrases, and two were words; in the LVS group, 132 were phrases, 10 were words, and three were clauses.

### 3.3. Temporal Adverbials in English–Chinese Translation

For the 24 temporal adverbials identified in the English source texts, the 35 learners translated them as temporal adverbials in 684 instances, as other types of adverbials in 10 instances, as non-adverbial expressions in 107 instances, and omitted them in 39 instances. Realization as a temporal adverbial in the translation was not significantly associated with either form or position in the English source texts, with detailed results presented in Table 11. Likelihood-ratio tests showed no significant overall association for form, *χ*^2^(2) = 2.07, *p* = 0.356, or position, *χ*^2^(2) = 0.26, *p* = 0.878. The sensitivity analysis excluding the single temporal adverbial occurring in medial position in the English source texts yielded the same conclusion, with no significant association for either form, *χ*^2^(2) = 1.92, *p* = 0.383, or position, *χ*^2^(1) < 0.01, *p* = 1.000.

**Figure 4:**
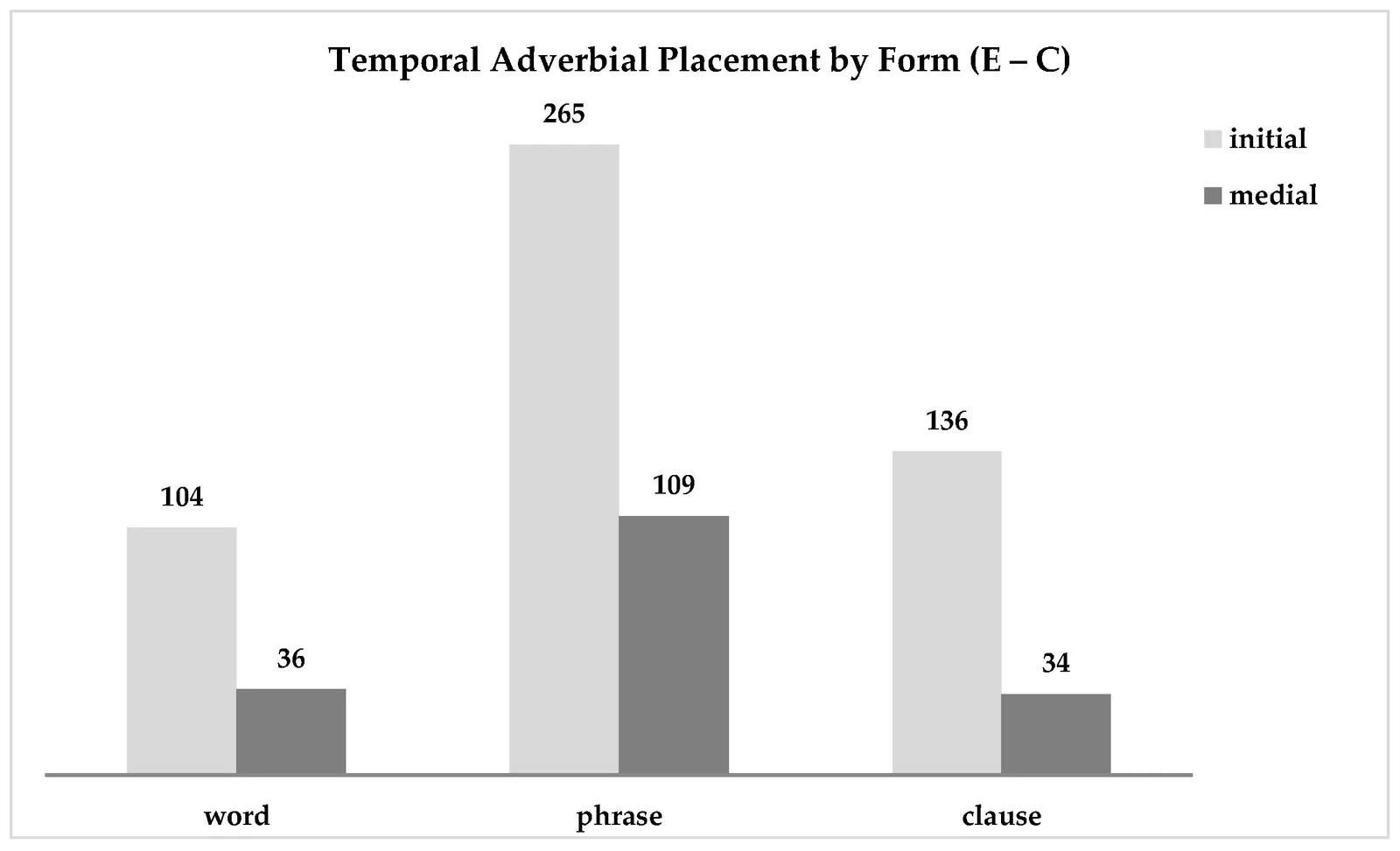
Distribution of temporal adverbials by form and position in English–Chinese translation.

Temporal adverbials in the Chinese translations occurred more frequently in initial than in medial position across all three forms (see Figure 4). Placement in the translation was not significantly associated with form in the translation, *χ*^2^(2) = 1.89, *p* = 0.388. Placement in the Chinese translations was also not significantly associated with either form, *χ*^2^(2) = 0.05, *p* = 0.975, or position, *χ*^2^(2) = 3.08, *p* = 0.215, in the English source texts. The sensitivity analysis excluding the single temporal adverbial occurring in medial position in the English source texts yielded the same conclusion, with no significant association for either form, *χ*^2^(2) = 0.05, *p* = 0.975, or position, *χ*^2^(1) = 2.91, *p* = 0.088.

### 3.4. Locational Adverbials in English–Chinese Translation

For the 15 locational adverbials identified in the English source texts, the 35 learners translated them as locational adverbials in 352 instances, as other types of adverbials in 39 instances, as non-adverbial expressions in 126 instances, and omitted them in eight instances. Realization as a locational adverbial in the translation was not significantly associated with position in the English source texts, *χ*^2^(1) = 0.01, *p* = 0.933. A parametric bootstrap likelihood-ratio test yielded the same result, *LR* = 0.01, *p* = 0.940. The association remained non-significant when the three English source texts were included as a categorical control variable, *χ*^2^(1) = 1.94, *p* = 0.163, and when the academic book preface was excluded, *χ*^2^(1) = 0.76, *p* = 0.384.

**Figure 5:**
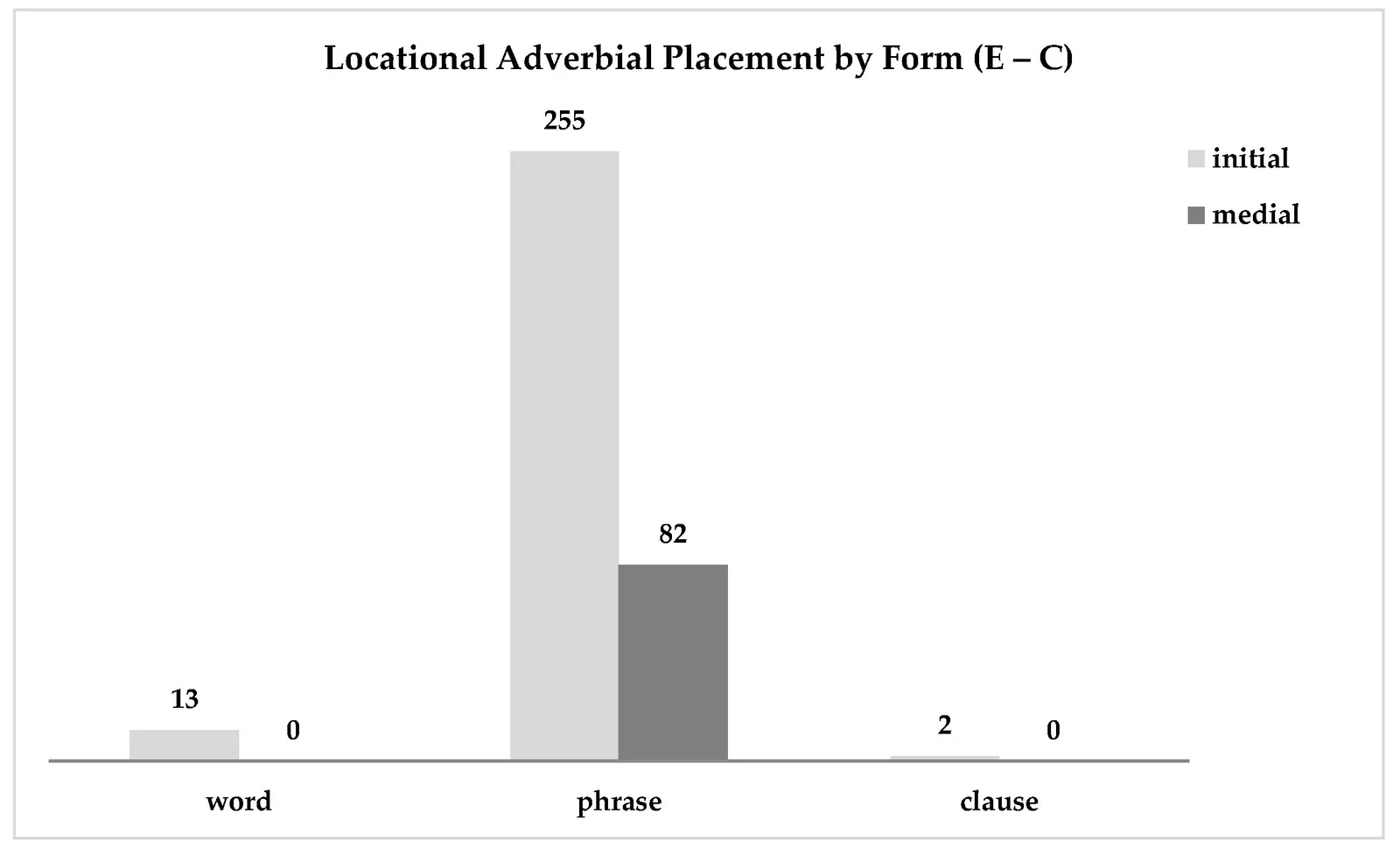
Distribution of locational adverbials by form and position in English–Chinese translation.

Of the 352 locational adverbials realized as locational adverbials in the translations, 270 occurred in initial position and 82 in medial position, with none occurring in final position. By form, 337 were phrases, of which 255 (75.67%) occurred in initial position and 82 (24.33%) in medial position. The remaining 13 words and two clauses all occurred in initial position (see Figure 5).

Placement in the Chinese translations was significantly associated with position in the English source texts, with detailed results presented in Table 12. A likelihood-ratio test confirmed a significant overall association, *χ*^2^(1) = 11.92, *p* < 0.001. Of the 227 locational adverbials occurring in initial position in the English source texts, 220 (96.92%) occurred in initial position in the translations and seven (3.08%) in medial position. Of the 125 occurring in final position in the English source texts, 50 (40.00%) occurred in initial position and 75 (60.00%) in medial position. A parametric bootstrap likelihood-ratio test also showed a significant association, *LR* = 11.92, *p* < 0.01. The association remained significant when the three English source texts were included as a categorical control variable, *χ*^2^(1) = 7.27, *p* < 0.01, and when the academic book preface was excluded, *χ*^2^(1) = 9.01, *p* < 0.01.

## 4. Discussion

The findings reveal several differences in the realization and placement of temporal and locational adverbials across the two translation directions. In Chinese–English translation, realization of temporal adverbials varied with form and position in the source text. Their placement differed by form in the translation and was also related to position in the source text. For locational adverbials, neither the realization nor the placement differed significantly between canonical sentences and sentences with LVS constructions. Within each sentence type, no significant associations were found with form or position in the source text. In English–Chinese translation, temporal and locational adverbials occurred only in initial or medial position when realized as the same adverbial type. Placement in the Chinese translations was significantly associated with position in the English source texts for locational adverbials, whereas no such association was found for temporal adverbials. The following sections discuss these findings in relation to the realization and distribution of adverbials in English and Chinese and the choices of placement made by the learners.

### 4.1. Temporal Adverbials in Chinese–English Translation

Learners did not always render temporal information as a temporal adverbial when translating from Chinese into English, but sometimes expressed it through other grammatical structures or omitted it. The realization of temporal adverbials in the Chinese source text as temporal adverbials in the English translation was associated with both their form and position in the Chinese source text. Compared with temporal words, phrases and clauses were more likely to be realized as temporal adverbials in English (*OR* = 16.06 and 4.73, respectively). Temporal adverbials in medial position in the Chinese source texts were less likely than those in initial position to be realized as temporal adverbials in English (*OR* = 0.26).

For temporal adverbials in the English translations, the descriptive distribution showed that learners tended to place temporal words in final position, whereas they placed phrases and clauses more often before the predicate. Placement differed across forms (*χ*^2^(2) = 9.33, *p* < 0.01), with Tukey-adjusted pairwise comparisons showing a significant difference between words and phrases but no significant differences between the other pairs. The GLMM indicated higher odds of pre-predicate placement for words than for phrases, contrary to the descriptive distribution. The descriptive proportions showed how often different forms occurred before the predicate across all translations, without considering which adverbials in the source texts they corresponded to. The GLMM, however, included random intercepts for individual adverbials in the source texts to account for the possibility that different source-text adverbials may have different placement distributions in their corresponding translations.

Placement of temporal adverbials in the English translations was significantly associated with position in the Chinese source text (*χ*^2^(2) = 21.75, *p* < 0.001), but not with form in the Chinese source text. Temporal adverbials that occurred initially in the Chinese source text were more likely to be placed before the predicate in English than those that occurred medially. This association remained significant in the sensitivity analysis excluding cases involving temporal adverbials in final position in the Chinese source text, suggesting that the result was not dependent on the inclusion of these cases.

Drawing on specific examples, the following section discusses the positional distributions of temporal words, phrases, and clauses in the English translations. It also considers how learners’ choices of placement relate to position in the Chinese source text and explores possible reasons for these choices.

#### 4.1.1. Temporal Words

Temporal words in the English translations differed from phrases and clauses in showing a preference for final position (61.84%). Final placement is one of the conventional options for temporal adverbs in English, although their distribution varies across lexical items and functions (Baker, 1986; Ćetković, 2014; Choi, 2025). The learners’ frequent choice of final position for temporal words can also be viewed in relation to the left-to-right expansion described for English by Liu (1992), with the main clause content preceding the temporal adverbial in linear order.

However, temporal words also commonly occurred before the predicate in the English translations, including “recently”, “immediately”, “then”, and “once”. Preverbal placement of these adverbs is also documented in English. Baker (1986) suggests that medial position is probably the most common for “recently”. Downing and Locke (2006) show that “immediately” can appear in initial, medial and final positions, with the choice of position related to focus. For “then”, Fox (1993) found clause-initial placement much more frequent than post-subject placement in written and spoken English, while Ćetković (2014) found post-subject placement frequent in English police reports. Hasselgård (2010) also provides corpus examples of “once” in medial position. These studies suggest that the preverbal placement of temporal words in the present corpus is also compatible with documented English usage.

The predominance of final placement, together with the preverbal use of particular temporal adverbs, may indicate that the word-level distribution reflects learners’ knowledge of the placement conventions associated with specific temporal adverbs in English rather than a uniform placement preference. Such item-specific knowledge may be particularly relevant when translating into English, where learners select among the positional options available for individual temporal adverbs.

#### 4.1.2. Temporal Phrases

Learners’ placement choices for temporal phrases in Chinese–English translations showed a more balanced distribution between initial (54.17%) and final positions (45.26%), with initial position appearing more frequently than final position. The medial position accounted for only 0.57%. Baker (1986) noted that adverbial phrases in English commonly appear at either the beginning or the end of a sentence. Klumm (2024) similarly found genre-related variation in English, with initial placement more frequent in commentaries and student stories, and final placement more frequent in news reports. Thus, the distribution of temporal phrases in the Chinese–English translations was broadly in line with previous descriptions of English temporal phrases, which commonly appear in both initial and final positions. One such structure is illustrated in Example 2.

(2)

Source text: **近些年来**它却渐成南京文化地标。

Pinyin: ***Jìnxiē niánlái*** tā què jiàn chéng Nájīng wénhuà dìbiāo.

Target text 1: ***In recent years***, it has gradually become a cultural landmark of Nanjing.

Target text 2: It has gradually become a cultural landmark of Nanjing ***in recent years***.

Of the 35 learners, 30 translated the source temporal expression as “in recent years”, with 27 placing the phrase in the initial position and three in the final. Both positions are acceptable in English. In this example, the predominance of initial position is in line with the overall GLMM finding that placement in the English translations was significantly associated with position in the Chinese source text, with temporal adverbials occurring initially in the source text more likely to be placed before the predicate than those occurring medially. Initial placement also allows the temporal framing of the Chinese source sentence to be maintained, in which the initial temporal expression sets the time span for the content that follows. When “in recent years” appears in initial position in English, it can perform the same function. Learners could therefore retain the source order and its frame-setting function while using a conventional English position. By contrast, the three translations with the temporal phrase in final position followed another conventional pattern in English. These translations presented the main content first and supplied the temporal information afterward. Thus, the two placements illustrate different ways of reorganizing the same temporal information within positions available in English, with initial placement more closely reproducing the organization of the source sentence.

Examples 3 and 4 provide a particularly informative comparison because they share the same subject and contain similar expressions, while the temporal phrases occur in different positions in the Chinese source sentences. In Example 3, where the temporal phrase preceded the subject in the Chinese source text, 32 corresponding English translations placed the phrase in initial position. However, in Example 4, where the temporal phrase appeared in medial position in the Chinese source text, only 16 corresponding English translations placed the phrase in initial position.

(3)

Source text: **1929年10月**，陈均沛在设计师李锦沛、赵深的介绍下，加入中国建筑师学会，成为中国近代执业建筑师。

Pinyin: ***1929 nián 10 yuè***, Chén Jūnpèi zài shèjìshī Lǐ Jǐnpèi, Zhào Shēn de jièshào xià, jiārù Zhōngguó Jiànzhùshī Xuéhuì, chéngwéi Zhōngguó jìndài zhíyè jiànzhùshī.

Target text: ***In October 1929***, Chen Junpei joined the Society of Chinese Architects and became a practicing architect in modern China under the introduction of Li Jinpei and Zhao Shen.

(4)

Source text: 陈均沛**于1929年3月**到南京国都设计技术专员办事处工作，任技士。

Pinyin: Chén Jūnpèi ***yú 1929 nián 3 yuè*** dào Nánjīng Guódū Shèjì Jìshù Zhuānyuán Bànshìchù gōngzuò, rèn jìshì.

Target text: Chen Junpei took office as an architect in the Nanjing Capital Designer Office ***in March 1929***.

The contrast may reflect different ways of presenting temporal information in the two Chinese sentences. In Example 3, the initial temporal phrase provides a temporal frame, whereas in Example 4, the date is integrated into the clause after the subject, with “*yú*” (于) explicitly marking the temporal relation. This distinction is in line with the contrast between pre-subject and post-subject temporal expressions in Chinese, with the former more closely associated with topicalization and temporal framing of the entire sentence (J. Y. Chen et al., 2025). The two examples therefore suggest that the influence of source-text position may involve more than the retention of linear order. When a temporal phrase occurs before the subject and serves a frame-setting function, retaining initial position in English can preserve both its position and its discourse function. When the temporal phrase occurs after the subject and is more closely integrated into the clause, learners may have greater flexibility to select another position available in English, including final position.

#### 4.1.3. Temporal Clauses

Temporal clauses showed the clearest preference for initial position among the three forms, with 69.11% appearing there. This distribution differs from the tendency of English temporal adverbial clauses to follow the main clause (Diessel, 2001, 2005; Ford, 1993; Klumm, 2024), but is consistent with Chinese, where temporal clauses commonly precede the main clause and establish a temporal frame (Chao, 1968; Wang, 2006; Yeh, 2000). Previous studies of English production by Chinese learners have reported a similar preference, with temporal clauses more often preceding the main clause (C. H. Chen, 2004; Y. Li & Wang, 2015; W. Li & Long, 2022). The learners in the present study likewise showed a preference for clause-initial placement in their English translations.

Temporal clauses in the Chinese source texts occurred only in initial or medial position, so position in the source text may also be relevant to their placement in English. Initial temporal clauses can establish a temporal frame before the main event is introduced. When learners retain this order in English, this frame-setting function can be preserved, as illustrated in Example 5.

(5)

Source text: **参赛队枪、弹在库期间**，枪弹库看护分队每两小时对枪弹库的门、窗、外围墙进行一次巡查。

Pinyin: ***Cānsàiduì qiāng, dàn zài kù qījiān***, qiāngdànkù kānhù fēnduì měi liǎng xiǎoshí duì qiāngdànkù de mén, chuāng, wàiwéiqiáng jìnxíng yí cì xúnchá.

Target text 1: ***When the teams’ guns and ammunition are in the warehouse***, the guards shall inspect the doors, windows and external walls of the warehouse every two hours.

Target text 2: The guards shall inspect the doors, windows and external walls of the warehouse every two hours ***when the teams’ guns and ammunition are in the warehouse***.

Both target texts are acceptable in English. Target text 1 retains the temporal clause before the main clause and therefore preserves the temporal framing of the Chinese source text. Target text 2 presents the main event first and adds the temporal clause afterward. The availability of both orders shows that initial position was not required by English grammar. The preference for initial position in this example therefore may reflect learners’ tendency to retain the order in the Chinese source text while preserving its temporal framing.

Clause placement is also related to the choice between “after-clauses” and “before-clauses”. The Chinese temporal clause marked by “*hòu*” (“after”) could be translated with an “after-clause” or reformulated with a “before-clause”. These alternatives can be realized with different orders of the two clauses, as illustrated in Example 6.

(6)

Source text: …**扫描确认枪支条形码后**，（参赛运动员）方可存取枪弹。

Pinyin: ***Sǎomiáo quèrèn qiāngzhī tiáoxíngmǎ hòu***, fāng kě cún qǔ qiāng dàn.

Target text 1: ***After scanning and confirming the bar code of the guns***, the athletes can store and withdraw the guns and ammunition.

Target text 2: They can store and retrieve the guns and ammunition ***after scanning and confirming the gun barcode***.

Target text 3: ***The barcode of the guns shall be scanned and confirmed before*** they fetch and store guns and ammunition.

Of the 35 translations, 21 used an “after-clause”, whereas four expressed the same temporal relation with a “before-clause”. The remaining ten did not use either type of clause and were therefore not included in this comparison. The massive use of “after” may be related to its direct correspondence with Chinese “*hòu*”. However, “*hòu*” (“after”) follows the temporal clause in Chinese, whereas English “after” introduces the clause. Jacob et al. (2024) suggest that serial lexical co-activation generates an initial sequence of translation equivalents that may subsequently be adjusted to meet target-language grammatical requirements. Learners using “after” in translation have two options. They could retain the clause order in the source text by placing “after” at the beginning of the temporal clause, or retain the subordinator between the two clauses by reversing the order of the two clauses. Target text 1 illustrates the first option. “After” appears at the beginning of the temporal clause, while the order of the two clauses remains the same as in the Chinese source text. Sixteen learners adopted this arrangement. Target text 2 illustrates the second option. The main clause appears first, and the after-clause follows it, reversing the clause order but keeping the temporal subordinator between the two clauses. Five learners adopted this arrangement. Thus, among the translations using an “after-clause”, learners more often retained the clause order in the source text than the position of the temporal subordinator between the two clauses. Target text 3 illustrates a different solution. By using “before”, learners could retain the clause order in the Chinese source text while keeping the temporal subordinator between the two clauses. However, this required the same temporal relation to be expressed from the opposite perspective, with the earlier event presented in the main clause and the later event introduced by “before”. Only four learners adopted this option, whereas 21 used an “after-clause”, indicating that learners more often chose the direct correspondence between “*hòu*” and “after” than reformulation with “before”.

The initial “after-clause” in Target text 1 and the before-clause in Target text 3 both present the “scanning and confirming of the barcode” before the “storage or withdrawal of the guns and ammunition”. By contrast, Target text 2, which contains a final “after-clause”, presents the two events in an order that differs from their chronological sequence. This difference can be interpreted in terms of sequencing iconicity, whereby events are expressed in the order in which they occur (Diessel, 2008). In Example 6, “scanning and confirming the barcode” precedes and serves as a prerequisite for “the storage or withdrawal of the guns and ammunition”. The order in Target texts 1 and 3 therefore corresponds not only to the chronological relation between the two events but also to the sequence described in the source text. In this example, the greater frequency of initial “after-clauses” than final ones may therefore be related to sequencing iconicity.

The strong preference for initial placement among temporal clauses shows a distribution resembling the front-weight tendency described for Chinese (Jin, 1998), rather than the end-weight tendency associated with English (Quirk et al., 1972, 1985). The contrast concerns the relative positioning of constituents of different length and syntactic complexity, with longer and more complex constituents tending to be placed toward the beginning of the clause in accordance with the front-weight tendency but toward the end in accordance with the end-weight tendency. Differences in the length and syntactic complexity of temporal adverbials may also be relevant to the form-related differences observed here. Temporal clauses typically contain more words and have more complex internal syntactic structures than temporal phrases or single words. In Chinese, syllabic length has been associated with whether temporal adverbs occur before or after the subject, whereas in English, the length of temporal adverbial clauses has been associated with whether they precede or follow the main clause (Yang, 2006; Diessel, 2008). Changing the position of a clause may also involve additional structural reorganization in translation. Previous studies have shown that word-order asymmetry can increase processing demands during translation (Ma et al., 2022; Ma & Li, 2024), suggesting that such reorganization may also be relevant to learners’ placement choices. However, these factors were not measured separately in the present study, making it difficult to assess how much they account for the differences among words, phrases, and clauses.

Overall, temporal adverbial placement in Chinese–English translation varied by form. Temporal words favored final position, phrases appeared in both initial and final positions, and clauses showed the strongest preference for initial position. Learners selected among the positions available in English rather than showing the same positional tendency across forms. Their choices were also associated with position in the Chinese source text.

### 4.2. Locational Adverbials in Chinese–English Translation

Locational adverbials in sentences with LVS constructions were less often realized as locational adverbials in the English translations than those in canonical sentences (35.71% vs. 59.52%). However, realization in the English translations was not significantly associated with sentence type in the Chinese source text, and the initial-only sensitivity analysis led to the same conclusion. The lower realization proportion in the English translations of sentences with LVS constructions therefore represents a descriptive tendency rather than a statistically supported difference between the two sentence types.

Learners’ choices of locational adverbial position were considered separately in translations from canonical sentences and sentences with LVS constructions because the two structures in the Chinese source texts differ in basic word order. In both groups, learners placed locational adverbials more often after the predicate, broadly corresponding to the common final placement of locational adverbials in English. The proportion of post-predicate placement was 53.10% in translations from sentences with LVS constructions and 60.00% in those from canonical sentences. However, placement in the English translations was not significantly associated with sentence type in the Chinese source text.

In a Chinese canonical sentence, the locational adverbial specifies where the event takes place. In a Chinese LVS construction, by contrast, the sentence first presents a location and then introduces the entity that exists or appears there, with the locative element before the predicate and the subject after it (Y. Chen & Jing-Schmidt, 2014). English also allows structures in which the locational adverbial occurs first and the entity is introduced later, such as an initial locational adverbial followed by a “there be” structure or locative inversion.

Among the translations of Chinese LVS constructions, 102 used a “there be” structure or its variants, indicating that this was a frequent choice among the learners. Locative inversion was also used, but it occurred less frequently. However, the use of a “there be” structure did not determine the position of the locational adverbial, which could appear either in initial or final position. These English structures provided learners with several ways of rendering Chinese LVS constructions, some of which retained the locative-first order, as illustrated in Example 7.

(7)

Source text: **在中山陵东北的小茅山顶**有一处古朴典雅的中式庭院。

Pinyin: ***Zài Zhōngshānlíng dōngběi de Xiǎomáo shān dǐng*** yǒu yí chù gǔpǔ diǎnyǎ de zhōngshì tíngyuàn.

Target text 1: There is a simple and elegant Chinese-style courtyard ***on the top of Xiaomao Mountain in the northeast of Sun Yat-sen’s Mausoleum***.

Target text 2: ***On the top of Xiaomaoshan Mountain in the northeast of Dr. Sun Yat-sen’s Mausoleum***, there is a simple and elegant courtyard in the Chinese style.

Target text 3: ***On the top of Xiaomao Mountain in the northeast of the mausoleum*** lies a simple and elegant Chinese-style courtyard.

Target text 4: ***In the northeast of Sun Yat-sen’s Mausoleum***, there is a courtyard with Chinese features ***on the top of Xiaomao Mountain***.

The four translations in Example 7 illustrate the different structural choices made by learners. Target text 1 uses a “there be” structure and places the locational adverbial at the end of the sentence. Target text 2 also uses a “there be” structure, but keeps the locational adverbial in initial position. Target text 3 places the locational adverbial first and uses locative inversion. Target text 4 separates the two parts of the location, placing the broader location first and the more specific location at the end. These translations show that learners could render Chinese LVS constructions through several English structures, with the locative-first order retained in some translations but not consistently.

Among these structural choices, locative inversion is particularly informative because it resembles Chinese LVS constructions not only in word order but also in how information is organized. Ojea (2020) describes English locative inversion as a non-canonical construction in which an initial locative expression sets the scene before the postverbal noun phrase introduces an entity into the discourse. Chinese LVS constructions likewise present the location first and then introduce the entity that exists or appears there (Y. Chen & Jing-Schmidt, 2014). Despite this close correspondence, locative inversion was used much less often than the “there be” structure in the present data. The less frequent use of locative inversion may indicate that learners favored a more conventional English construction over an option that more closely preserved the word order and information organization of the Chinese LVS construction.

In terms of form, the locational data showed limited variation. Translations from canonical sentences included 123 phrases and two words, whereas translations from sentences with LVS constructions included 12 words, 130 phrases, and only three clauses. This limited range of forms is also understandable given that locational adverbials in Mandarin are commonly realized as locative phrases, particularly “*zài* + location” structures (J. Y. Fan, 1982; Y. Xu, 2022). The uneven distribution of these forms limits the extent to which their placement can be meaningfully compared. The locational data therefore provide a weak basis for drawing conclusions about form-related differences in placement.

### 4.3. Temporal Adverbials in English–Chinese Translation

Of the 840 translations corresponding to the 24 temporal adverbials in the English source texts, 684 were translated as temporal adverbials. Whether a temporal adverbial in the English source texts was translated as a temporal adverbial in Chinese was not significantly associated with either its form or its position, suggesting that these two characteristics did not systematically shape learners’ realization choices.

All 684 temporal adverbials in the Chinese translations occurred before the predicate, in line with previous findings that Chinese temporal adverbials generally occur in preverbal positions (Yang, 2006; W. Li, 2012; Wang, 2006). The initial position was more frequent than the medial position for all three forms, accounting for 74.29% of words, 70.86% of phrases, and 80.00% of clauses. The mixed-effects model showed no significant association between form and placement in the Chinese translations, providing no evidence of differences in placement across the three forms.

Placement of temporal adverbials in the Chinese translations was not significantly associated with either form or position in the English source texts. This provides no evidence that learners’ overall placement choices for temporal adverbials in the Chinese translations were systematically associated with these characteristics in the English source texts. Although translated Chinese may retain structural traces of English source texts (Xiao, 2010, 2015), the present data did not provide evidence of such structural retention in temporal adverbial placement. This is particularly evident for temporal adverbials occurring in final position in the English source texts, which were repositioned before the predicate when retained as temporal adverbials in the Chinese translations. Example 8 illustrates this reorganization.

(8)

Source text: Make the effort to learn a few words of Mandarin ***before your trip***.

Target text: **在启程前**，努力学几句普通话。

Pinyin: ***Zài qǐchéng qián***, nǔlì xué jǐ jù pǔtōnghuà.

In Example 8, “before your trip” is translated as an independent temporal adverbial and appears in initial position as “*zài qǐchéng qián*” (before setting off). In this position, the phrase establishes the temporal frame within which the following action, learning a few words of Mandarin, is to take place. This use of initial position is consistent with the frame-setting function of sentence-initial temporal expressions in Chinese (Wang, 2006). The repositioning illustrated in Example 8 is in line with previous studies reporting similar repositioning of temporal adverbials in English–Chinese translation (G. Xu, 2002; X. Zhang, 2008; Z. Zhou, 1995). The translations of “before your trip” also varied in how the reference event was expressed. Some learners used expressions such as “*lǚxíng*” (trip) and “*chūxíng*” (traveling), whereas others narrowed the reference to the point of departure through “*qǐchéng*” (set off) or “*chūfā*” (depart). Other translations made the destination explicit, as in “*qù Zhōngguó*” (go to China), or specified the type of trip as “*chūchāi*” (business trip). These choices show that positional adjustment could be accompanied by lexical-semantic adjustment. The English expression “trip” leaves the reference event relatively broad, while learners selected among more specific ways of expressing it in Chinese to match the context. Translating the temporal adverbial therefore involved not only repositioning it within Chinese word order but also deciding how the reference event should be represented. This variation may reflect the lack of a one-to-one lexical-semantic correspondence between the English expression and its Chinese alternatives, which may have encouraged learners to make the reference event more explicit in Chinese or reformulate it to fit the context.

### 4.4. Locational Adverbials in English–Chinese Translation

Of the 525 translations corresponding to the 15 locational adverbials in the English source texts, 352 were translated as locational adverbials. Among the 173 translations not realized as locational adverbials, the locational information was reformulated as non-adverbial expressions or other types of adverbials in 165 translations and omitted in eight. Subjects accounted for 95 of the 126 non-adverbial realizations, including 82 translated from the recurring “In Chapter X” expressions. These expressions in the academic book preface represented 26.7% of the locational adverbials in the English source texts but accounted for 49.7% of the reformulated cases in the Chinese translations. Despite this concentration of reformulations, locational adverbials occurring in initial and final positions in the English source texts did not differ significantly in their realization as locational adverbials in Chinese. Realization in the Chinese translations was still not significantly associated with position in the English source texts after excluding the English source text taken from an academic book preface.

The 352 locational adverbials in the Chinese translations all occurred before the predicate, with 270 in initial position and 82 in medial position. This distribution accords with the typical preverbal placement of Chinese locational adverbials (J. Y. Fan, 1982; Y. Xu, 2022). The distribution of form was highly uneven, with phrases accounting for 337 of the 352 locational adverbials, compared with 13 words and two clauses. The small numbers of words and clauses limit meaningful statistical comparison of placement across forms.

Locational adverbial placement in the Chinese translations was significantly associated with position in the English source texts, unlike its non-significant association with realization. This association also remained significant after the academic book preface was excluded. This suggests that position in the English source texts was related to placement rather than realization, specifically to learners’ choice between initial and medial positions in the Chinese translations. Locational adverbials in initial position in the English source texts were overwhelmingly retained in initial position in the Chinese translations, whereas those in final position in the English source texts occurred more often in medial position. Of the translations corresponding to locational adverbials in initial position in the English source texts, 96.9% used initial position in Chinese. For locational adverbials in final position, the corresponding translations used initial position in 40.0% of cases and medial position in 60.0%. Example 9 illustrates these two placement options in the corresponding Chinese translations. Among the 34 translations, five occurred in initial position, while 29 occurred in medial position.

(9)

Source text: Building up a good relationship is important to conduct business ***in China***.

Target text 1: 在中国，建立友好关系对做生意至关重要。

Pinyin 1: ***Zài Zhōngguó***, jiànlì yǒuhǎo guānxì duì zuò shēngyì zhìguān zhòngyào.

Target text 2: 建立良好的关系对**在中国**经商很重要。

Pinyin 2: Jiànlì liánghǎo de guānxì duì ***zài Zhōngguó*** jīngshāng hěn zhòngyào.

In Target text 1, “in China” is translated as “*zài Zhōngguó*” in initial position, where it establishes a spatial frame for the proposition that follows. In Target text 2, the same locational expression occurs in medial position and directly modifies “*jīngshāng*” (conduct business). This contrast is in line with J. Y. Fan’s (1982) observation that an initial “*zài* + location” phrase tends to establish a spatial frame, whereas medial position more directly specifies the location of the action. The predominance of medial placement in these translations may be related to the close association between “in China” and “conduct business” in the English sentence.

Although locational adverbials in final position in the English source texts occurred more often in medial position overall, this distribution was not found in every case. Example 10 shows a case in which initial position was more frequent than medial position in the Chinese translations.

(10)

Source text: Business cards are an absolute must ***in China***.

Target text 1: **在中国**，名片是绝对需要的。

Pinyin 1: ***Zài Zhōngguó***, míngpiàn shì juéduì xūyào de.

Target text 2: 名片**在中国**是必需品。

Pinyin 2: Míngpiàn ***zài Zhōngguó*** shì bìxūpǐn.

In Target text 1, “in China” is translated as “*zài Zhōngguó*” in initial position, where it establishes the spatial frame for the statement that follows. In Target text 2, the same locational phrase appears in medial position, between the subject and the predicate. In contrast to Example 9, “in China” in Example 10 is not closely associated with a separate action such as “conduct business.” Instead, it sets the location for the whole statement that “business cards are essential”. The contrast may also be related to the structure of the English sentences. In Example 9, “in China” forms part of the infinitival expression “to conduct business in China,” and most learners kept the locational expression close to “*jīngshāng*” (conduct business) in Chinese. In Example 10, “in China” appears at the end of the sentence rather than as part of an infinitival expression, and initial placement was more frequent than medial placement.

Taken together, the two examples suggest that sentence structure may help account for variation in learners’ choices between initial and medial positions within the broader tendency for locational adverbials occurring finally in the English source texts to appear medially in Chinese.

## 5. Conclusions

This study investigated how advanced Chinese learners of English realized and placed temporal and locational adverbials in Chinese–English and English–Chinese translation. In C-E translation, learners did not always translate temporal adverbials as such in English, with realization as temporal adverbials being more frequent for phrases and clauses than for words and also more frequent for those occurring initially rather than medially in the Chinese source texts. In the English translations, learners placed temporal words in final position most often, while phrases were distributed mainly between initial and final positions and clauses occurred most often initially. For locational adverbials in C-E translation, the proportion of locational adverbials in the Chinese source texts that were realized as locational adverbials in the English translations was lower for LVS constructions than for canonical sentences, but realization was not significantly associated with sentence type in the Chinese source texts. Locational adverbials in the target texts occurred more often after the predicate in both groups, with no significant difference between the two sentence types. In E-C translation, all temporal and locational adverbials in the target texts occurred before the predicate. Locational placement between initial and medial positions differed according to position in the English source texts, whereas temporal placement was not significantly associated with either form or position in the English source texts. When locational adverbials occurred initially in the English source texts, learners strongly favored initial placement in the Chinese translations, whereas for those occurring finally in the English source texts, the corresponding locational adverbials in the Chinese translations occurred more often in medial position. Some case-specific variation was nevertheless observed.

Learners made their placement choices within the positional options available in the target language. Position in the source texts therefore did not map directly onto placement in the translations, and its relationship with placement was not consistent across adverbial types and translation directions. This positional adjustment was particularly evident in E-C translation, where temporal and locational adverbials occurring finally in the English source texts were repositioned before the predicate when realized as adverbials in Chinese. Beyond these positional adjustments, learners sometimes changed the grammatical realization of adverbial information, reformulating it through other adverbial and non-adverbial structures in both translation directions. In E-C translation, such changes in realization sometimes extended beyond grammatical structure to lexical-semantic reformulation, with some reference events expressed more explicitly or reformulated to fit the context.

By examining realization and placement in both translation directions, the study characterizes how advanced Chinese learners of English realize and place temporal and locational adverbials in translation. Considering realization and placement together provides a more detailed description of learners’ choices across adverbial types and translation directions. Future studies using closely matched source materials across directions and larger learner samples could further examine the stability of these distributions across comparable translation tasks and proficiency levels.

## Figures and Tables

**Table 1:** Annotation tags for adverbial type, position, and form.

**Layer**	**Category**	**Tag**
Adverbial type	Temporal adverbial	tadv
	Locational adverbial	sadv
Position	Initial	initial
	Medial	medial
	Final	final
Form	Temporal word	t
	Temporal phrase	tp
	Temporal clause	tc
	Locational word	s
	Locational phrase	sp
	Locational clause	sc

**Table 2:** Distribution of annotated expressions by form and position in the source texts.

Direction	Type	Word	Phrase	Clause	Total
C→E	Temporal	5/11/0	13/7/2	12/6/0	56
	Locational, canonical	-	3/3/0	-	6
	Locational, LVS	7/0/0	5/0/0	-	12
E→C	Temporal	0/1/2	5/0/11	3/0/2	24
	Locational	-	10/0/5	-	15

Note. Values are reported in the order initial/medial/final.

**Table 3:** GLMMs for realization and placement of temporal adverbials in C-E translation.

Model	DV	Predictor(s)
Realization	Realization (same-type/other)	Form in the source text (word/phrase/clause) Position in the source text (initial/medial/final)
Placement 1	Placement (before/after the predicate)	Form in the translation (word/phrase/clause)
Placement 2	Placement (before/after the predicate)	Form in the source text (word/phrase/clause) Position in the source text (initial/medial/final)

**Table 4:** GLMMs for realization and placement of locational adverbials in C-E translation.

Model	DV	Predictor(s)
Realization 1	Realization (same-type/other)	Sentence type in the source text (canonical/LVS)
Realization 2 (LVS)	Realization (same-type/other)	Form in the source text (word/phrase)
Realization 3 (canonical)	Realization (same-type/other)	Position in the source text (initial/medial)
Placement 1	Placement (before/after the predicate)	Sentence type in the source text (canonical/LVS)
Placement 2 (canonical)	Placement (before/after the predicate)	Position in the source text (initial/medial)
Placement 3 (LVS)	Placement (before/after the predicate)	Form in the source text (word/phrase)

**Table 5:** GLMMs for realization and placement of temporal adverbials in E-C translation.

Model	DV	Predictor(s)
Realization	Realization (same-type/other)	Form in the source text (word/phrase/clause) Position in the source text (initial/medial/final)
Placement 1	Placement (initial/medial)	Form in the translation (word/phrase/clause)
Placement 2	Placement (initial/medial)	Form in the source text (word/phrase/clause) Position in the source text (initial/medial/final)

**Table 6:** GLMMs for realization and placement of locational adverbials in E-C translation.

Model	DV	Predictor(s)
Realization	Realization (same-type/other)	Position in the source text (initial/final)
Placement	Placement (initial/medial)	Position in the source text (initial/final)

**Table 7:** Mixed-effects logistic regression of the realization of temporal adverbials on form and position in the source text.

	*β*	*SE*	*OR*	95% CI for *OR*	*p*	
**Intercept**	1.500	0.545	4.48	[1.54, 13.03]	0.006	**
**phrase vs. word**	2.776	0.619	16.06	[4.77, 54.04]	<0.001	***
**clause vs. word**	1.554	0.604	4.73	[1.45, 15.47]	0.010	*
**medial vs. initial**	−1.336	0.503	0.26	[0.10, 0.71]	0.008	**
**final vs. initial**	−0.021	1.483	0.98	[0.05, 17.93]	0.989	

Note. * *p* < 0.05; ** *p* < 0.01; *** *p* < 0.001.

**Table 8:** Mixed-effects logistic regression of placement of temporal adverbials on form in the translation.

	*β*	*SE*	*OR*	95% CI for *OR*	*p*	
**Intercept**	1.996	0.799	7.36	[1.54, 35.26]	0.013	*
**phrase vs. word**	−1.970	0.663	0.14	[0.04, 0.51]	0.003	**
**clause vs. word**	−1.559	0.747	0.21	[0.05, 0.91]	0.037	*

Note. * *p* < 0.05; ** *p* < 0.01.

**Table 9:** Sensitivity analysis using mixed-effects logistic regression of placement of temporal adverbials on form and position in the source text.

	*β*	*SE*	*OR*	95% CI for *OR*	*p*	
**Intercept**	1.222	0.956	3.39	[0.52, 22.11]	0.201	
**phrase vs. word**	0.815	1.044	2.26	[0.29, 17.48]	0.435	
**clause vs. word**	0.807	1.068	2.24	[0.28, 18.17]	0.450	
**medial vs. initial**	−3.142	0.851	0.04	[0.008, 0.23]	<0.001	***

Note. *** *p* < 0.001.

**Table 10:** Mixed-effects logistic regression of realization of locational adverbials on sentence type in the source text.

	*β*	*SE*	*OR*	95% CI for *OR*	*p*
**Intercept**	0.614	0.628	1.85	[0.54, 6.33]	0.328
**LVS vs. canonical**	−1.335	0.767	0.26	[0.06, 1.18]	0.082

**Table 11:** Mixed-effects logistic regression of realization of temporal adverbials on form and position in the English source texts.

	***β***	***SE***	***OR***	**95% CI for *OR***	***p***
**Intercept**	1.480	1.638	4.39	[0.18, 108.88]	0.366
**phrase vs. word**	1.419	1.481	4.13	[0.23, 75.32]	0.338
**clause vs. word**	0.168	1.708	1.18	[0.04, 33.62]	0.922
**medial vs. initial**	−1.182	2.452	0.31	[0.003, 37.51]	0.630
**final vs. initial**	−0.056	0.936	0.95	[0.15, 5.92]	0.953

**Table 12:** Mixed-effects logistic regression of placement of locational adverbials on position in the English source texts.

	***β***	***SE***	***OR***	**95% CI for *OR***	***p***	
**Intercept**	4.826	1.087	124.71	[14.81, 1050.18]	<0.001	***
**final vs. initial**	−5.547	1.449	0.0039	[0.0002, 0.0667]	<0.001	***

Note. *** *p* < 0.001.

## Data Availability

The data are not publicly available due to privacy and ethical reasons.

## Abbreviations

The following abbreviations are used in this manuscript:

MTI	Master of Translation and Interpreting
C-E	Chinese–English
E-C	English–Chinese
SVO	Subject–Verb–Object
RL	right-to-left
LR	left-to-right
LVS	Locative–Verb–Subject
CCL	Center for Chinese Linguistics
BLCU	Beijing Language and Culture University
ESL	English as a second language
CET-6	College English Test Band 6
GLMM	Generalized Linear Mixed-Effects Model
